# Supplementary material for: Ionising Radiation Induces Promoter DNA Hypomethylation and Perturbs Transcriptional Activity of Genes Involved in Morphogenesis during Gastrulation in Zebrafish
Source: Int J Mol Sci. 2020 Jun 4;21(11):4014. doi: 10.3390/ijms21114014 (PMC7312202; doi:10.3390/ijms21114014)
Supplement: Supplementary file 1 [file ijms-21-04014-s001.zip › Supplementary Informations.docx]

**Supplementary Figure S1:** Assessment of biological variability of RNAseq samples by hierarchical clustering. (**a**) Dendrogram obtained by hierarchical cluster of all samples generated by RNAseq. Potential outliers (red) were removed to increase the power of the subsequent differential analysis. (**b**) Dendrogram obtained by hierarchical cluster of the 37 selected samples used for the differential expression analysis. For each sample, the irradiation batch is indicated (I13, I14, I30, I32), as well as the replicate number and the dose rate used for exposure (C: non exposed control, 50mG: 50 mGy/h, 5mG: 5 mGy/h, 0.5mG: 0.5 mGy/h, 50microG: 0.05 mGy/h, 5microG: 0.005 mGy/h).

**Supplementary Figure S2:** Enrichment of biological pathways deregulated in 6 hpf embryos after exposure to IR at 0.005, 0.05, 0.5, 5 and 50 mGy/h. (**a**) Heat map of representative zebrafish GO pathways. Dose rates are indicated at the bottom in mGy/h. Colours indicate the p-values from Fisher’s exact test. (**b**) Dot plot of KEGG enrichment pathways using human gene orthology. Dose rates are indicated at the bottom in mGy/h. Note that no KEGG pathway was found to be enriched in the condition 0.05 mGy/h due to the small number of DEG. The total numbers of DEG in the KEGG pathways selected on the dot plot are indicated in brackets. Colours indicate the enrichment p-values from Fisher’s exact test and dots size is proportional to the number of DEG in the given pathway.

**Supplementary Figure S3:** Volcano plot displaying the differential expression (fold change and adjusted p-value) of genes annotated in the zebrafish genome with the GO term (**a**) “mitochondrion” (GO:0005739) and (**b**) “Response to oxidative stress” (GO:0006979) at 50, 5 and 0.5 mGy/h. DEG (|fold change| ≥ 1.5 and adjusted p-value (padj < 0.01) are represented in red and yellow colours.

**Supplementary Figure S4:** Dot plot of zebrafish GO term enrichment using the 589 DEG found in common at 5 and 50 mGy/h. Colours indicate the enrichment p-values from Fisher’s exact test and dots size is proportional to the number of genes constituting the given pathway.

**Supplementary Figure S5:** Transcription factor binding sites enrichment in the promoters of DEG at (**a**) 50 mGy/h (**b**) 5 mGy/h and (**c**) 0.5 mGy/h. Blue dot indicates significant enrichment of the DNA binding site (Z-score > 6 and Fisher-score > 3).

**Supplementary Figure S6:** Dot plot of differential methylation levels of transposon (indicated as DNA TP) and retrotransposons (SINE and LINE elements) at (**a**) 50 mGy/h and (**b**) 5 mGy/h. The % of methylation differences are shown in a window of 2 kb up and down the transposon (start and end indicated by the dashed lines). A minor fraction of cytosine is differentially methylated (fraction < 0.005) as indicated at the top of each dot plot (blue: hypomethylated compared to control, red: hypermethylated).

**Supplementary Figure S7:** Assessment of biological variability of WGBS samples by hierarchical clustering.

**Supplementary Table T1:** Complete list of zebrafish GO terms enriched in the RNAseq data at 0.005, 0.05, 0.5, 5 and 50 mGy/h. The p-values from the Fisher’s exact test are indicated.

**Supplementary Table T2:** List of deregulated genes involved in the retinoic acid signalling. The log2(fold change) and adjusted p-values of all genes annotated in the zebrafish genome with the GO terms “regulation of retinoic acid receptor signalling” (GO:0048385), “response to retinoic acid” (GO:0032526) and “retinoic acid receptor signalling pathway” (GO:0048384) are displayed. Significantly regulated genes in the RNAseq data are indicated in bold (adjusted p-value < 0.01).

**Supplementary Table T3:** Table of TF binding sites enriched significantly (Z-score > 6 and Fisher’s score > 3) in the promoter of genes deregulated in the RNAseq data at 50, 5 and 0.5 mGy/h. The Z-scores and Fisher-scores obtained from oPOSSUM are indicated for each TF binding sites. The last two columns indicate respectively if the given TF binding sites was enriched in the three conditions and expressed in gastrula embryos (based on Zfin expression patterns, http://zfin.org/).

**Supplementary Table T4:** Results of MCNPX simulations and operational dosimetry obtained with radio photoluminescent dosimeter (mGy/h) at the target dose rates of 50, 5, 0.5, 0.05 and 0.005 mGy/h (sd: standard deviation to the mean).

**Supplementary Table T5:** Quality control of sequencing reads of the 45 biological samples analysed by RNAseq. Replicates number, dose rate, irradiation batch and developmental stage are indicated for each sample, as well as the reads number obtained and the % of reads with Phred score Q > 30 (R1: read1, R2: read2).

**Supplementary Table T6:** Quality control of bisulfite conversion rate of the 12 samples analysed by WGBS. Replicates number, dose rate, irradiation batch and developmental stage are indicated for each sample, as well as the % conversion of C to T.

**Supplementary Table T7:** Quality controls of sequencing reads of the 12 samples analysed by WGBS. Replicates number, dose rate, irradiation batch and developmental stage are indicated for each sample, as well as the reads number obtained and the % of reads with Phred score Q > 30.
