# Supplementary material for: Ionising Radiation Induces Promoter DNA Hypomethylation and Perturbs Transcriptional Activity of Genes Involved in Morphogenesis during Gastrulation in Zebrafish
Source: Int J Mol Sci. 2020 Jun 4;21(11):4014. doi: 10.3390/ijms21114014 (PMC7312202; doi:10.3390/ijms21114014)
Supplement: Supplementary file 1 [file ijms-21-04014-s001.zip › Supplementary_Figures.pdf]

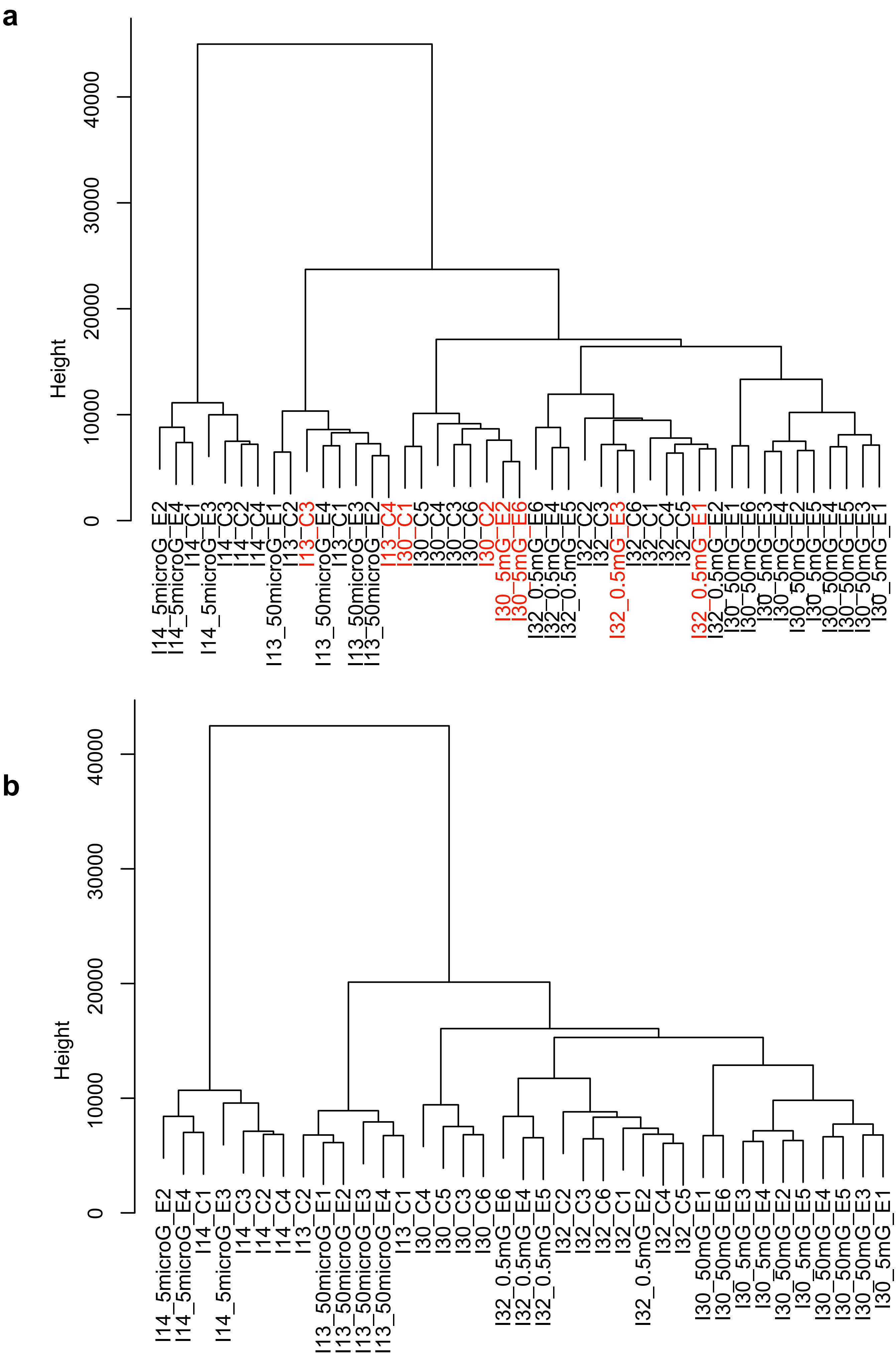

Supplementary Figure S1

a

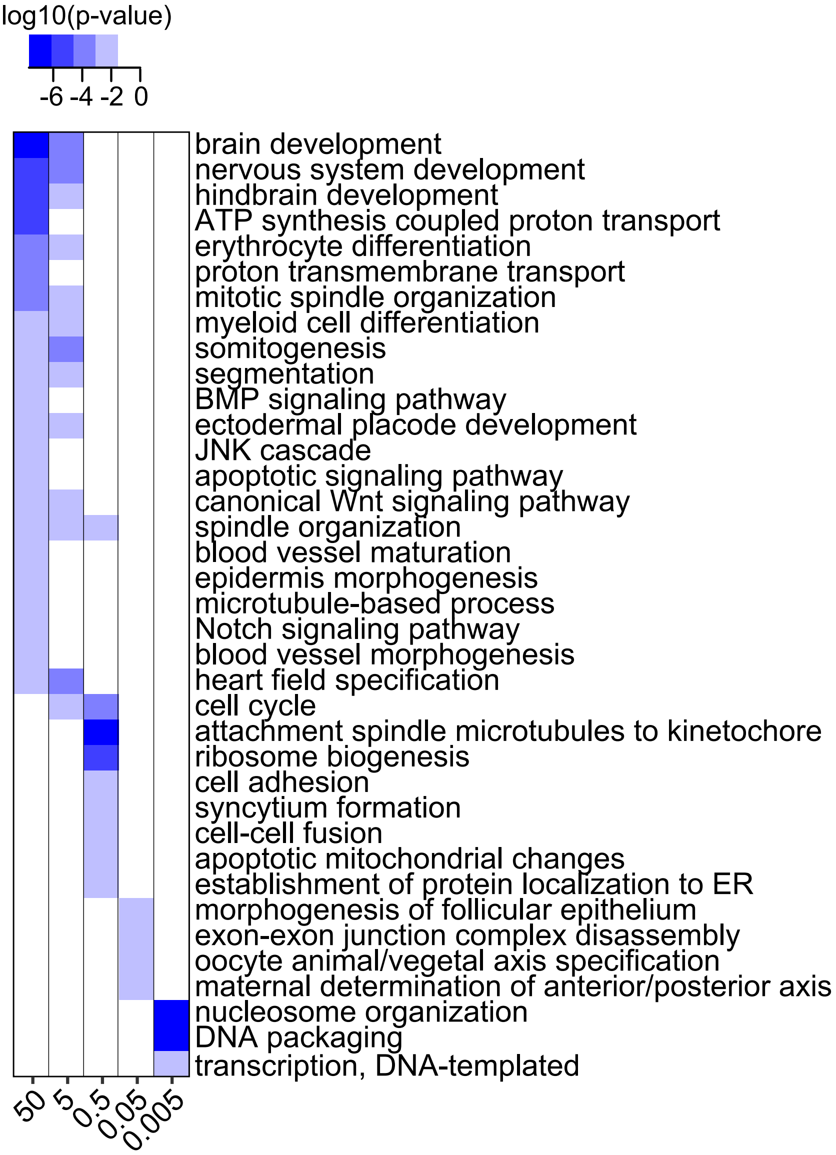

b

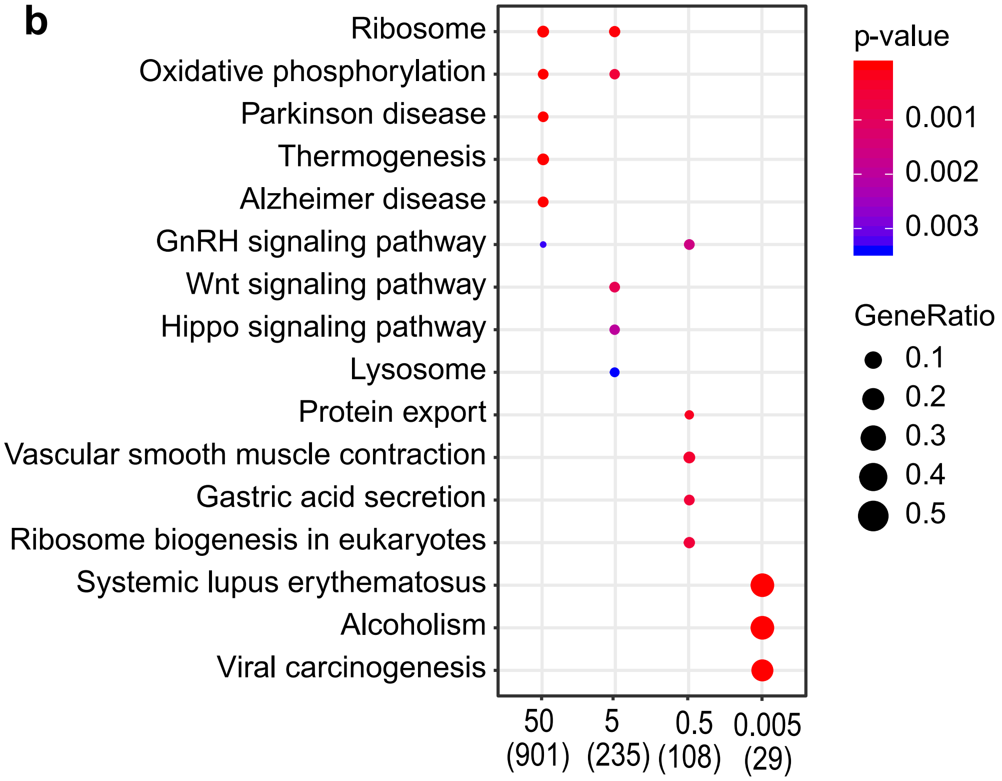

Supplementary Figure S2

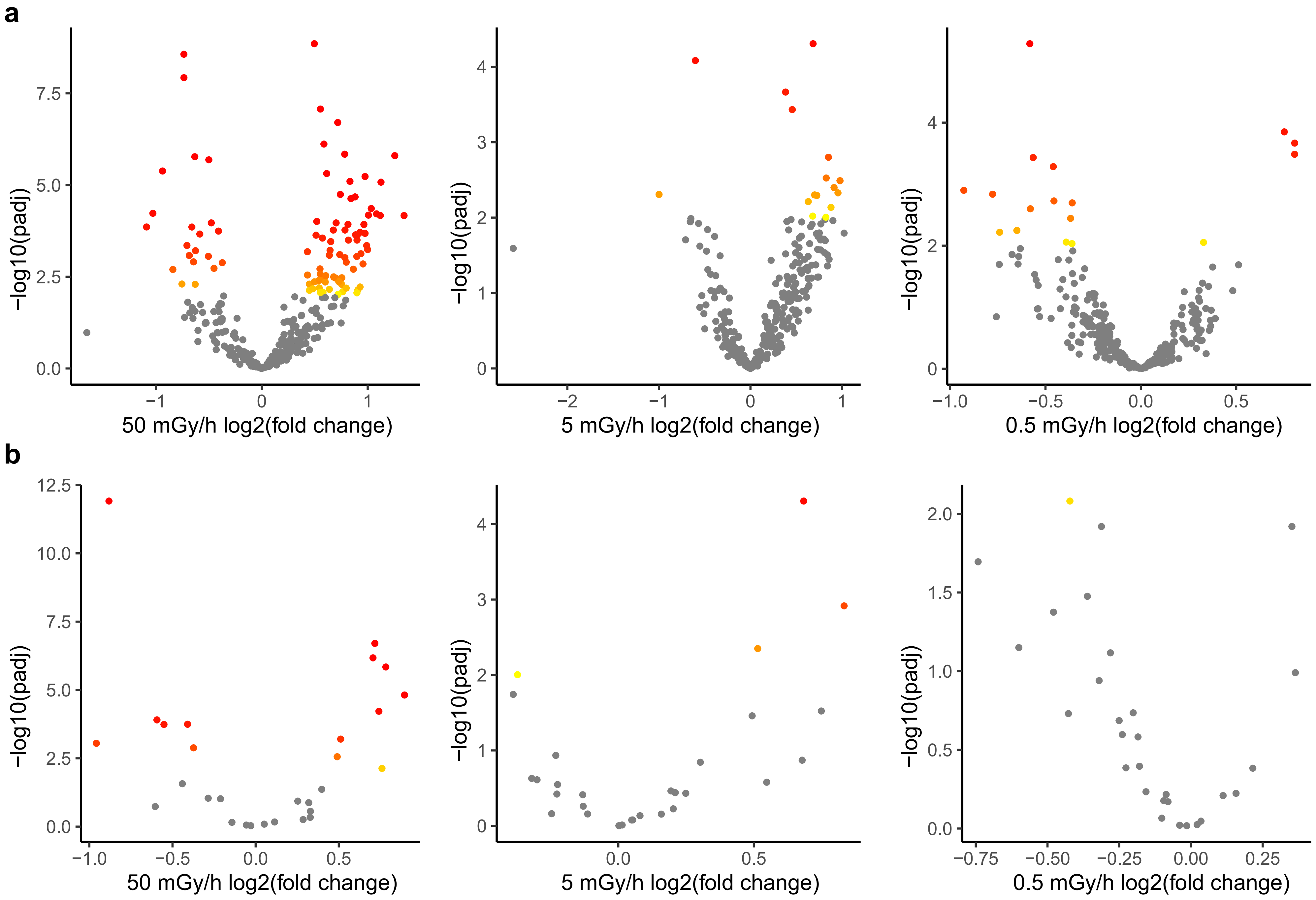

Supplementary Figure S3

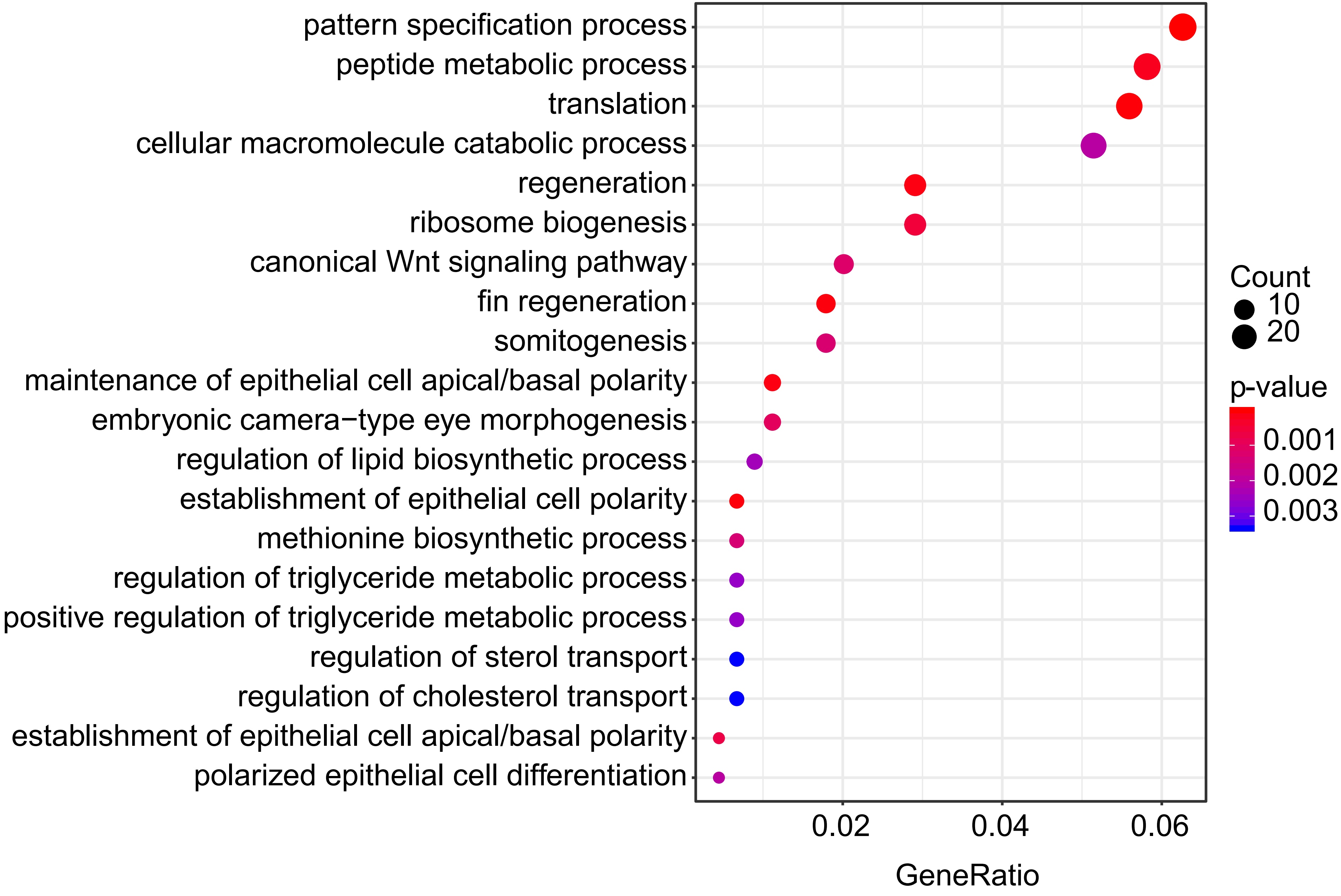

Supplementary Figure S4

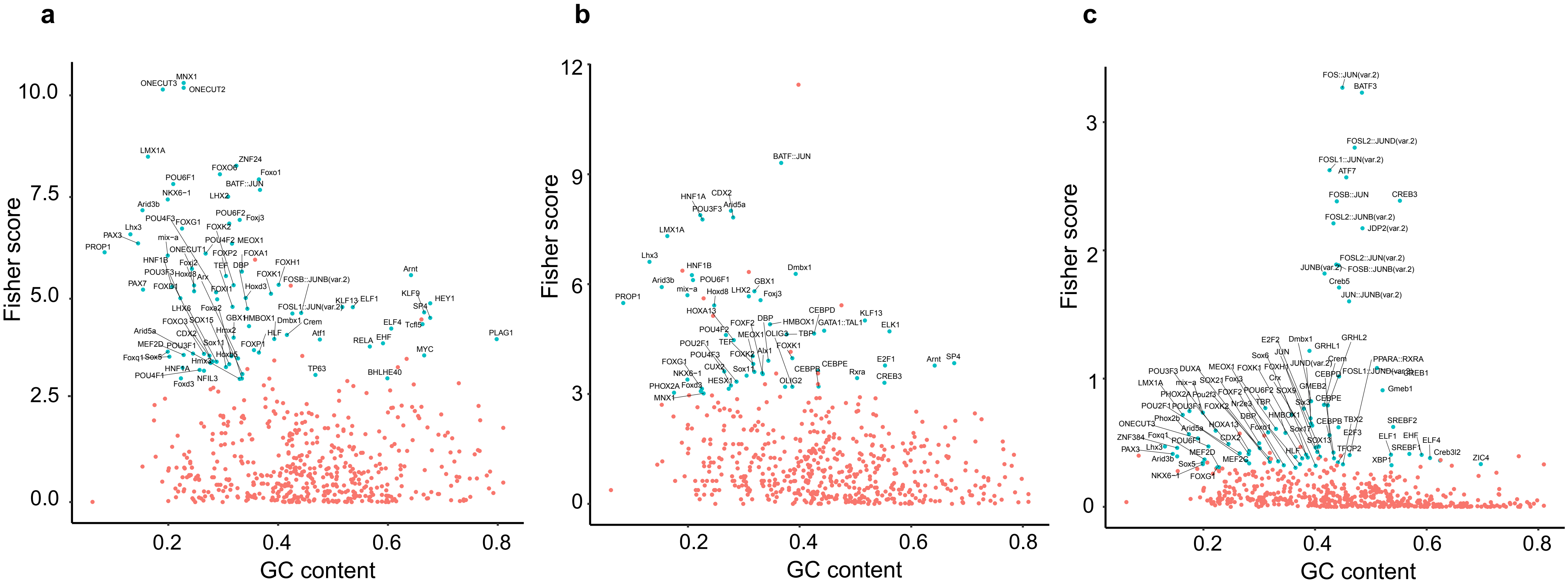

Supplementary Figure S5

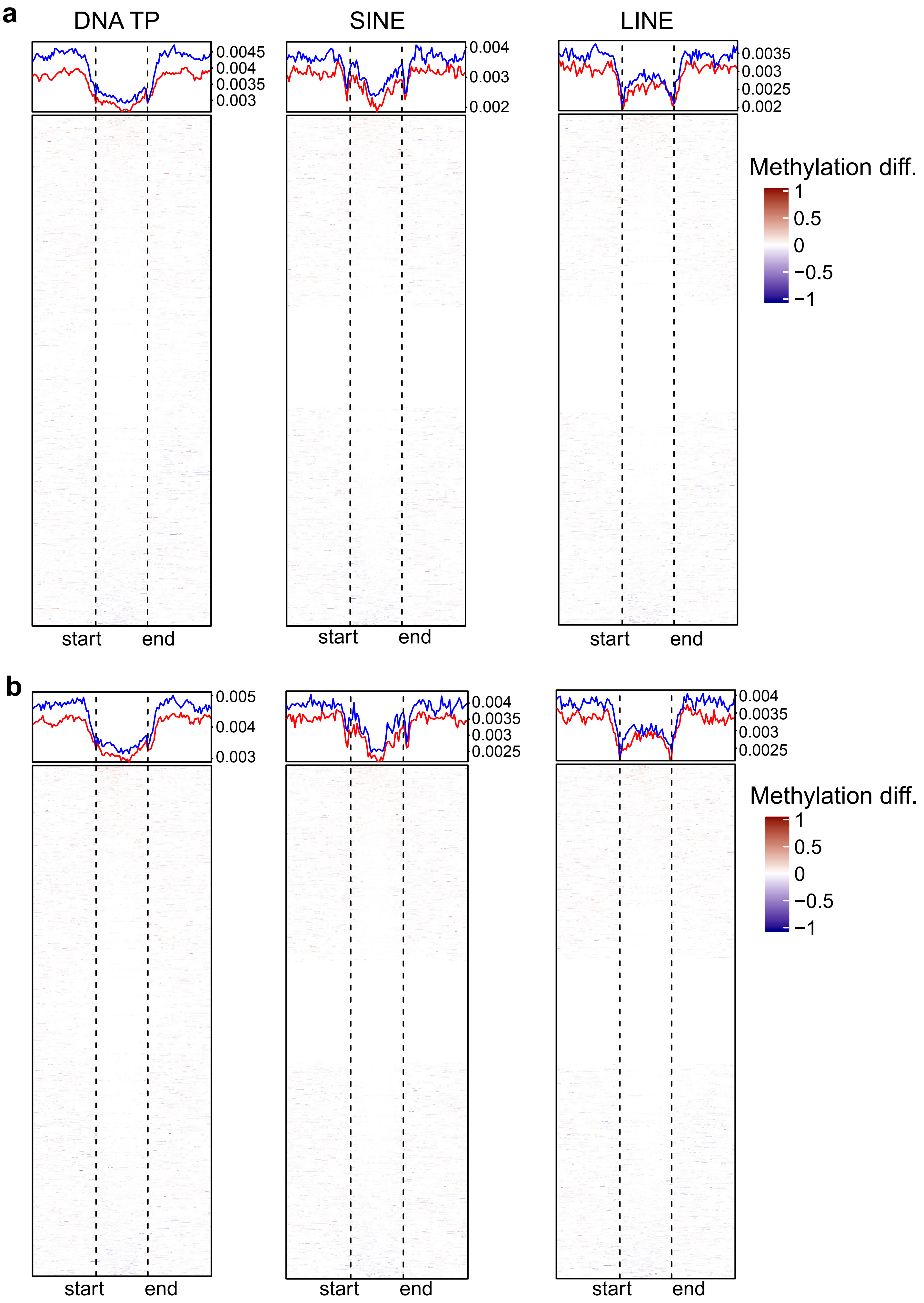

Supplementary Figure S6

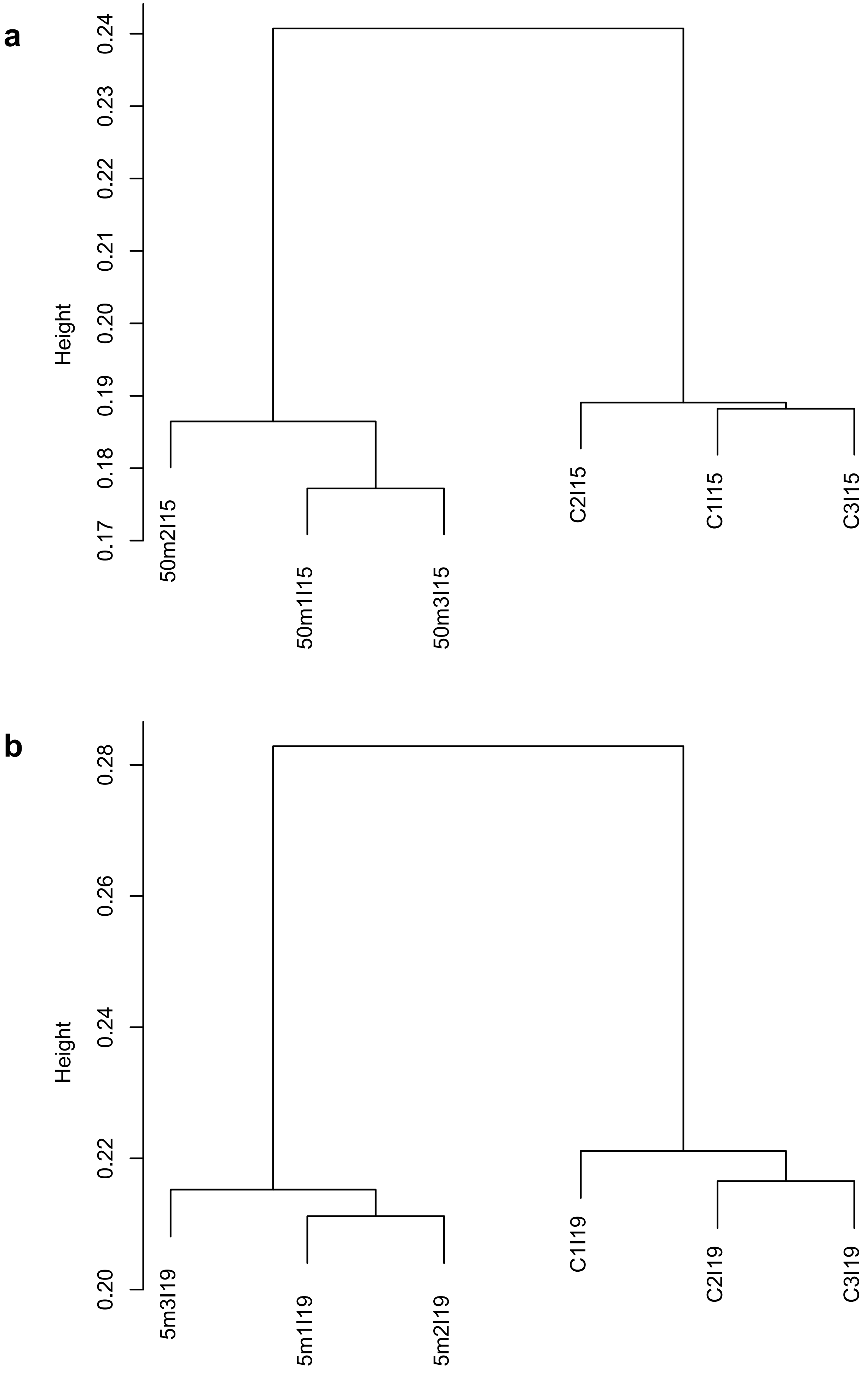

Supplementary Figure S7
